# An integrated approach to the analysis of antioxidative peptides derived from Gouda cheese with a modified β-casein content

**DOI:** 10.1038/s41598-022-17641-x

**Published:** 2022-08-03

**Authors:** Anna Iwaniak, Damir Mogut, Piotr Minkiewicz, Justyna Żulewska, Małgorzata Darewicz

**Affiliations:** 1 Chair of Food Biochemistry, Faculty of Food Science, University of Warmia and Mazury in Olsztyn, Pl. Cieszyński 1, 10-719 Olsztyn-Kortowo, Poland; 2 Department of Dairy Science and Quality Management, Faculty of Food Science, University of Warmia and Mazury in Olsztyn, Oczapowskiego 7, 10-719 Olsztyn-Kortowo, Poland

**Keywords:** Peptides, Proteins, Biochemistry, Proteolysis, Databases

## Abstract

This study is the first to present an integrated approach involving in silico and in vitro protocols that was pursued to analyse an antioxidative potency of Gouda cheese with modified content of β-casein. Firstly, the predictions of the presence of antioxidant peptides in the casein sequences were computed using the BIOPEP-UWM database. Then, the antioxidative bioactivity of six variants of Gouda cheese (with reduced, normative, and increased content of β-casein at the initial and final stage of ripening) was assessed. Finally, the RP-HPLC–MS/MS was applied to identify antioxidative peptides in Gouda-derived water-soluble extracts (WSEs). Analyses were supported with the heatmaps and the computation of parameters describing the efficiency of proteolysis of caseins in the modified Gouda cheeses, i.e., the frequency and the relative frequency of the release of antioxidative fragments during cheese ripening (A_Eexp_ and W_exp_., respectively). All Gouda cheese variants exhibited the antioxidative potential which differed depending on the assay employed. The highest antioxidative activity (ABTS^**·**+^ radical scavenging effect, FRAP, and Fe-chelating) was observed for WSEs derived from Gouda cheese with increased content of β-casein after the 60th day of ripening. The results obtained suggest the potential of Gouda cheese as the antioxidant-promoting food.

## Introduction

Since 1950s, when the biological role of casein-derived peptides had been mentioned for the first time, loads of information were published concerning the discovery of new peptides, their function, identification, and production^1^. Today, it is well-known that peptides derived from foods exhibit various bioactivities, and thus are considered as biologically active regulators^2^.

Gouda is one of the most popular and known cheeses worldwide^3^ and, according to scientific reports, it was discovered as the source of enzyme inhibitors like: angiotensin-converting enzyme (ACE; EC 3.4.15.1) and/or dipeptidyl peptidase IV (DPP-IV; EC 3.4.14.5), responsible for blood pressure and glucose level regulation, respectively^4,5^. Although, some studies have confirmed the antioxidative potential of ripened cheeses^6^, there is paucity of literature data concerning specific types of cheeses as sources of peptidic antioxidants. To the best of our knowledge, no data can be found about the antioxidative peptides identified in Gouda cheese.

According to Sołowiej et al.^7^, casein—being a major cheese protein, is often incorporated to foods due to its certain functional properties, like e.g., consistency and fat-emulsifying ability. When looking at the issue entitled “peptides in ripened cheeses”, loads of data show that many peptides featuring various bioactivities were found in β-casein (β-CN) using in silico and in vitro protocols^8–10^.

In silico analyses became a supportive tool to study proteins and biopeptides of food origin because of the rapid development of methods for data collection and tools aiding the analysis of molecules. It especially concerns databases and software that support the analysis of the potential of bioactive peptides and their protein sources^11^. This methodology of peptide analysis is less costly and time-consuming when compared to the classical approach relying briefly on the following methodological steps in studying biopeptides from food sources: (i) finding a promising protein source of biopeptides and enzyme to hydrolyze it; (ii) isolating peptide(s) from a hydrolysate using, e.g. chromatography methods; and (iii) evaluating the bioactivity of isolated peptide(s)^12^. Therefore, scientists try to combine bioinformatic- and classical-assisted approaches to get a deeper insight into the nature of biologically active peptides. This methodology is called an integrated approach (a hybrid approach)^13^. This term was introduced by Udenigwe^14^ as the alternative solution for dealing with problems related to, e.g., identification of peptides in the protein by supporting the research with the information found in databases.

Taking into account the following aspects: (1) the abundance of β-casein in the fragments with biological activity; (2) the lack of sufficient data on the potential of Gouda cheese as the source of peptidic antioxidants, and (3) the growing popularity of methods involving bioinformatics to study proteins and peptides originating from foods, this study aimed to apply an integrated approach to analyze water-soluble extracts (WSEs) derived from Gouda cheese with a modified content of β-casein as the source of antioxidant peptides.

## Results and discussion

### Prediction of the presence of antioxidant peptides in casein sequences (in silico analysis)

The presence of antioxidant peptides in caseins was predicted using the parameter called the profile of potential biological (i.e. antioxidative) activity of protein. Briefly, it shows what antioxidative peptides match the protein sequence (see “Methods”). Such profiles were acquired from BIOPEP-UWM database^15^. The BIOPEP-UWM database described by Minkiewicz et al.^15^ is a collection of peptides with dozens of bioactivities that were identified experimentally by researchers. Moreover, this database is a universal tool serving for the evaluation of proteins as the potential sources of peptides^15^.

Citing the words of Halliwell^16^ “The terms ‘antioxidant’, ‘oxidative stress’ and ‘oxidative damage’ are widely used but rarely defined”. Thus, Tirzitis and Bartosz^17^ tried to systemize some terms concerning the so-called antioxidant activity of molecules. They have emphasized that there is a difference between antiradical and antioxidant activity of a compound and these terms do not coincide. Generally, antiradical bioactivity refers to a molecule’s ability to react with free radicals, whereas antioxidant activity entails the inhibition of the oxidation processes. Therefore, the tests involving a free radical, like e.g., DPPH and/or ABTS, provide the data concerning the radical scavenging activity or antiradical activity of a compound^17^. Thus, regardless of the test applied (e.g., FRAP, DPPH^.^, ABTS^.+^, hydroxyl radical etc.), peptides with these bioactivities are specified in the BIOPEP-UWM database as “antioxidative”. Additional reason for such “definition” of the activity of these peptides is the measure of bioactivity. The BIOPEP-UWM database “accepts” the bioactivity of any peptide expressed as IC_50_ or EC_50_ (concentration of a peptide needed to inhibit or exhibit the effect corresponding to its half-maximal activity, respectively). The precise information on the measure as well as the individual sequence bioactivity was provided in the link called “Additional information”^15^. The results of bioinformatic predictions of the occurrence of antioxidative motifs in the casein sequences are shown in Table 1S (Supplementary Materials). These results include the predictions made for β-CN as well as other casein fractions like α_s_ and κ. It results from the applied approach involving further identification of antioxidative peptides in Gouda cheese-derived samples.

Although the most common genetic variants of β-CN occurring in taurine breeds are A_1_, A_2_, and B^18^, antioxidative peptides were reported in all genetic variants β-CN. Some antioxidative peptides: KVLPVPQK, VLPVPQK, YQEP, and YQEPVLGP, occurred only in B variant of β-casein. There is no information about antioxidative activity of corresponding fragments of other genetic variants of the above protein, not only in the BIOPEP-UWM^15^, but also in the EROP-Moscow^19^ and MBPD databases^20^.

Among β-caseins, the best source of antioxidative peptides was genetic variant B (18 sequences encrypted), whereas the other variants of this protein fraction contained 14 sequences each. The number of peptide fragments found in a protein sequence (i.e. the higher, the better) suggests the high probability for their enzymatic release from a protein^21^. Looking at the length of the chain of antioxidative peptides encrypted in β-caseins, they were composed of two to maximum 11 amino acid residues. The most abundant were dipeptides < tripeptides < tetrapeptides. Longer motifs were less dominant in caseins. The length of a peptide chain affects the match of a protein sequence (the shorter the peptide is, the greater is its chance to match the protein)^8^.

Moreover, we analyzed the potential regularities between antioxidative motifs encrypted in β-caseins that may determine antioxidative activity. They were rich in Phe (F), His (H), Leu (L), and Tyr (Y). According to Zambrowicz et al.^22^, Y is found as an amino acid eliciting the antioxidative effect. Udenigwe and Aluko^23^ applied Partial Least Square (PLS) modelling to study quantitative structure–activity relationships (QSAR) of antioxidative peptides derived from food protein hydrolysates. They found that the presence of some amino acids, like Trp (W), Y, Met (M), C (Cys), H, F, and Pro (P), may contribute to the antioxidative activity of both purified food-derived and synthetic natural peptides. The presence of H negatively affected the antioxidative function of peptides^23^. However, the cited authors concluded that the presence of this residue in synthetic peptides promoted the antioxidative activity in a linoleic acid oxidation model. No such effect was observed in DPPH and superoxide radical-scavenging models^23^. Another QSAR studies confirmed that the presence of C-terminal W, Y and/or C enhanced the antioxidative activity of tripeptides. This was the case with the dataset of 19 peptide sequences representing i.a. DPPH-scavenging and FRAP activities^24^. N-terminal amino acids, like W or F, and Ile (I), M and Val (V) at the middle position were advantageous to the antioxidant bioactivity of tripeptides. In contrast, this activity was suppressed by the following residues: Ala (A), Gly (G), Ser (S) at position 1 and Asn (N), Asp (D), and C at position 2, but only in the case of the DPPH-scavenging effect^24^. To summarize, the presence of antioxidative peptides in the casein sequences, their variety in length of the chains, and their amino acid composition showing the antioxidative potential of β-caseins were the prerequisites to continue the studies on “casein-rich foods”, like different variants of Gouda cheese.

### Monitoring of the proteolysis of Gouda cheese variants using SDS-PAGE electrophoresis

The designed Gouda cheese-making process consisted of a series of the filtration steps, i.e., MF and UF, carried out at different temperatures, which resulted in different contents of β-CN in cheese milk used to produce cheeses with the modified β-CN content (Table 1). The β-CN content was determined at 0.85% for control milk (Table 1). St-Gelais and Hache^25^ reported similar results, who determined the β-CN content at 0.83% in control milk as well as at 0.95, 1.08 and 1.22% in three milks supplemented with different amounts of β-CN. It should be noted that in the present study, the increase in β-CN content was not as high as expected. However, any changes in αs-CN to β-CN ratio affected not only the coagulation process of milk but also the course of proteolysis of the produced cheese. In the cheese-making process, more than 90% of casein is retained in the curd^26^. In a study carried out by Hallen et al.^26^ with model cheeses, the retention of particular casein fractions in cheese curd was determined at 93.3 ± 10.3, 97.7 ± 8.6, 92.7 ± 9.8 and 93.6 ± 7.7 for α_s1_-CN, α_s2_-CN, β-CN and κ-CN, respectively.

**Table 1:** The content of β-casein (β-CN) in cheese milk (M) used to produce Gouda cheese with the modified content of β-casein: normative (M-CN^0^), increased (M-CN^+^), and reduced (M-CN^−^). ^a,b*,*c^Statistically significant differences between the mean values within the same row.

	M-CN^0^	M-CN^+^	M-CN^−^
%β-CN/CN(SDS-PAGE)	31.93^b^	35.65^a^	22.11^c^
CN (%)	2.67^a^	2.59^a^	2.62^a^
% β-CN	0.85^b^	0.92^a^	0.58^c^

The ratios of α_s_-CN to β-CN in cheeses were determined on day 1 to monitor changes in the proteolysis process during cheese ripening (Table 2). However, no significant (P ≥ 0.05) differences were detected in the proportion of α_s_-CN to β-CN between cheeses with normative (G-CN^0^) and increased (G-CN^+^) content of β-CN. It is worth noting that β-CN content of milk used to manufacture these cheeses varied^27^ (Table 1). Further studies are needed to understand the coagulation process and properties of rennet gel produced from milk with the altered ratio of α_s_-CN/β-CN. The use of para-κ-CN as an internal standard allowed determining the percentage degradation of α_s_-CN and β-CN. No difference (P ≥ 0.05) was detected in α_s_-CN % degradation between cheeses with normative (G-CN^0^) and decreased (G-CN^−^) content of β-CN. The extent of α_s_-CN degradation in the cheese with the increased β-CN content was lower (P < 0.05) than in the other cheeses. St-Gelais and Hache^25^ produced cheeses with an increased β-CN content and found out that α-casein was hydrolyzed during cheese ripening and that the rate of its degradation decreased as protein and β-casein concentrations increased in the cheese. de Roos et al.^28^ and Dunnewind et al.^29^ have shown that the addition of a small amount of β-CN to the κ-CN/chymosin solution resulted in a strong suppression of chymosin association. β-CN competes with chymosin for one or more binding sites located on para-κ-CN, or a potential binding site for chymosin on the para-κ-CN molecule is shielded by β-CN. St-Gelais and Hache^25^ assumed that β-CN could also shield a potential binding site for chymosin on the α-CN in enriched cheeses. Generally, any changes in the ratio α-CN to β-CN would modify the properties of milk and resulted cheese. Van Hekken and Holsinger^30^ concluded that milk gels produced from milk enriched with β-casein formed softer gels more prompt to syneresis and lower water holding capacities than skim milk gels.

**Table 2:** The ratio of α_s_-casein (α_s_-CN) to β-casein (β-CN) in Gouda on day 1 and percentage degradation of α_s_-casein and β-casein after 60 days of ripening of Gouda cheeses produced with normative (G-CN^0^), increased (G-CN^+^), and reduced (G-CN^−^) content of β-casein. ^a,b^Statistically significant differences between the mean values within the same row.

	G-CN^0^	G-CN^+^	G-CN^−^
α_s_-CN/β-CN ratio on day 1	1.21^b^	1.07^b^	1.62^a^
α_s_-CN % degradation	69.71^a^	44.47^b^	64.52^a^
β-CN % degradation	59.04^a^	64.97^a^	66.49^a^

### Water-soluble extracts (WSEs) of Gouda cheeses and their antioxidative activity

Four assays were used to assess the antioxidative effect of WSEs. According to the literature, there are several methods for quantifying the antioxidant bioactivity of a molecule^31,32^. Such a variety of methods results from the lack of a “quantification pattern” to determine this bioactivity due to the different oxidation mechanisms. Thus, to make any statement, it is recommended to employ at least two different methods to determine the antioxidative effect of a system^33^. To date, the antioxidant potential of cheeses of different types and origins has been measured using tests involving DPPH radical, ABTS cation radical, Fe ion-chelators, and ferric ion-reductants^6,34,35^. Thus, we used the same assays to measure the antioxidant capacities of WSEs derived from Gouda cheese variants (see Table 3). Moreover, being guided by words of Dontha^36^, according to which “an antioxidant is a molecule capable of inhibiting the oxidation of another molecule”, we converted the obtained results into IC_50_ values.

**Table 3:** Antioxidative activity of WSEs derived from Gouda cheeses with a modified content of β-casein. G-CN^0^_1_, G-CN^0^_60_, G-CN^+^_1_, G-CN^+^_60_, G-CN^−^_1_, G-CN^−^_60_—Gouda cheese with normative, increased, and reduced content of *β*-casein after the 1st and 60th day of ripening (subscripts: 0 and 60, respectively).

	Assay	WSE derived from:					
		G-CN^0^_1_	G-CN^0^_60_	G-CN^+^_1_	G-CN^+^_60_	G-CN^−^_1_	G-CN^−^_60_
IC_50_ (mg/mL)	ABTS^**·**+^	7.804	6.808	7.627	5.948	5.962	6.212
	DPPH	10.750	8.971	7.203	8.962	7.830	9.515
	FRAP	2.183	2.062	2.287	1.647	1.684	2.379
	Fe-chelating	0.734	0.513	0.592	0.513	0.655	0.533

There were no statistically significant differences between antioxidative bioactivity of WSEs derived from different variants of Gouda cheese. According to Meira et al.^34^, who studied antioxidant activity of WSEs derived from ovine cheeses, it is difficult to establish the inter-relationships between the results of antioxidative activities. This may stem from various methods deployed that indicate the presence of various peptides in the WSEs but the activity is related to their different mode of action. Moreover, the following factors affect the antioxidant power of peptides: amino acid composition, size, amount and configuration (i.e. exposure of the terminal amino groups), and concentration of free amino acids^34^. Regardless of the test applied to measure the antioxidative effect of WSE, all samples exhibited this bioactivity. The highest effect was observed in the Fe-chelating ability of WSEs (the lowest values of IC_50_ parameter). Among them, WSE derived from freshly produced Gouda cheese (1st day; with normative content of β-casein) and from cheese after 60 days of ripening (Gouda with increased content of β-casein) displayed the highest Fe-chelating ability (IC_50_ for both cheese variants was 0.513 mg/mL). Öztürk and Akin^37^ analyzed the Fe-chelating potential of Turkish Tulum cheese during different ripening periods. They observed that Tulum WSEs had a higher Fe-chelating capacity than Roquefort, Cerrillano, and Pecorine-type cheeses produced from sheep milk. The highest bioactivity of Tulum cheese was observed on the 60th day of the ripening^37^. These findings are consistent with our study results concerning the samples derived from all variants of Gouda cheese. It can be explained by parallelism between ripening period and the formation of oligopeptides. Depending on the duration of the ripening process, plasmin, endogenous and microbial proteases are involved in the production of large and intermediate sized peptides from caseins. Then, secondary microflora leads to the transformation of these peptides to shorter fragments and amino acids^37^. The impact of hydrolysis time on Fe-chelating power was also confirmed for ovine milk caseinates hydrolyzed by microbial proteases^38^. During cheese proteolysis, the structure of proteins is disrupted, which increases solvent accessibility of amino acids which can chelate ions^39^. According to Meira et al.^33^, the metal-chelating function involves, i.a., amino acids possessing a ring (F, Y, H). Corrêa et al.^38^ suggested that residues, like C and H, were not hidden and exhibited redox and metal chelating abilities. Among 18 peptides identified in this experiment (see Table 2S in Supplementary Materials), 14 contained F, Y or H residues. Apart from peptides listed in Table 2S, also these containing phosphoserine residues may be involved in sequestering metal cations^34^. In turn, Huma et al.^40^ found that the presence of Q (Gln), K (Lys), M, Y, H, C, and P in peptide sequences determined the antioxidative effect of WSE of Roquefort cheese. 17 out for the 18 peptides identified using MS/MS in our WSE samples derived from Gouda cheese contained at least one of the above amino acid residues. Matemu et al.^41^ reported that some individual amino acids (Y, M, H, K, P, W) also possessed the antioxidative properties and/or influenced the antioxidant activity of peptides (see above). Moreover, when analyzing the other measures of the antioxidant power of the WSEs of all cheese samples, those derived from Gouda cheese with an increased content of β-casein on the 60th day of ripening had the highest FRAP (IC_50_ = 1.647 mg/mL) and elicited the strongest ABTS^·+^ radical scavenging effect (IC_50_ = 5.948 mg/mL). The comparison of the samples of the cheeses with the same content of β-casein and different ripening periods led to observe that regardless of the test applied, the antioxidative effect of “normative cheeses” increased over the ripening time (their IC_50_ values decreased). This regularity was observed in the samples of Gouda with a higher β-casein content, excluding the results of DPPH-radical scavenging activity (IC_50_ slightly changed from 7.203 to 8.962 mg/mL). In the case of Gouda cheese with a reduced β-casein content, the enhancement of antioxidant bioactivity between ripening periods was reported only for the Fe-chelating effect (IC_50_ changed from 0.655 to 0.533 mg/mL).

Perna et al.^33^ observed an increase in the FRAP of ovine ripening cheeses throughout the progression of the ripening, which was in agreement with our results concerning the WSEs from Gouda cheeses with normative and increased contents of β-casein except WSEs derived from the cheese with a lower β-casein content (IC_50_ changed from 1.684 to 2.379 mg/mL). In turn, Bottesini et al.^42^ reported a stable trend in the antioxidative capacity in Parmigiano Reggiano cheeses over the ripening period. These authors claimed that molecules responsible for antioxidant bioactivity (i.e. peptides and proteins) were not affected by biochemical processes taking place during cheese maturation. The side chains of amino acids have been suggested to maintain the same (or almost the same) antioxidant capacity as both components of peptides and free compounds^42^.

Although it can be said that all Gouda cheese samples exhibited antioxidant bioactivity; generally, it cannot be concluded that the modification of β-casein content caused a definitive increase of this bioactivity as the ripening process proceeded. It was dependent on the assay applied to measure the antioxidant power. Some of our results were in agreement with those obtained by other authors. For example, in the case of two cheese variants (four WSE samples; see Table 3), ABTS^·+^ test results showed an increase in the antioxidant bioactivity during cheese ripening. Revilla et al.^6^ analyzed 224 samples of cheeses with varying proportions of cow, ewe, and goat milks used for their production. They observed that the antioxidant effect of cheeses tested with the ABTS^·+^ method increased along with ripening time^6^. According to Revilla et al.^6^, the enhanced antioxidant effect of cheese samples during ripening was due to the progress in proteolysis, leading to the production of peptides and sulfur-containing amino acids, being well known antioxidants. Other studies into the antioxidant power of cheeses discussed by Revilla et al.^6^ revealed that the content of water-soluble peptides increased during ripening and reached the maximum in the first month of this process. Meira et al.^34^ studied the antioxidant potential of ovine ripening cheeses involving all tests that were also applied in this research. The results that were expressed as percentages of inhibition differed. This difference can be explained by, e.g., different scavenging patterns (mechanisms) for DPPH and ABTS radicals. The ABTS water-soluble pre-formed cation is built by oxidation of ABTS^.+^ with potassium persulfate. It is reduced in the presence of hydrogen-donating and chain-breaking antioxidant molecules. In turn, DPPH^.^ accepts an electron or hydrogen to become a stable diamagnetic molecule. Because DPPH^.^ is pre-dissolved in alcohol, thus it may not diffuse to target peptides present in an aqueous solution^34^. Moreover, higher concentrations of the samples in DPPH^.^ assay than in ABTS^·+^ test were applied, which could also indicate that the latter was a more sensitive method to assess the antioxidative activity of cheese-derived water-soluble extracts^34^.

To summarize, the highest antioxidative activity (ABTS^**·**+^ radical scavenging effect, FRAP, Fe-chelating) was observed for WSEs derived from Gouda cheese with increased content of β-casein after the 60th day of ripening. However, our results concerning the antioxidative potential of different WSEs varied depending on the assay applied. According to Shahidi and Zhong^32^, many assays were developed to measure the antioxidant capacity of food at the molecular and cellular levels. Each test has its pros and cons^32^. According to Chen et al.^43^, different antioxidant power assessment tests applied to the same WSE sample cause its diversified behavior dependent on the structure of the radical that reacts differently with the antioxidative molecule present in the WSE. Thus, taking into account the data concerning the antioxidant activity of WSEs derived from six variants of Gouda cheese, we decided to continue our studies with the identification of antioxidant peptides in the cheese samples.

### Identification of antioxidant peptides of WSEs from Gouda cheese with a modified content of β-casein

The results of RP-HPLC–MS/MS identification of antioxidant peptides in all WSE variants, including the peptide sequence, its protein source, retention time, and mass-to-charge ratio, are shown in Table 2S. Eighteen sequences of antioxidant peptides were identified in WSEs. Nine peptides (LHS, YYV, GTQY, YQLD, FYQL, YQKFP, AVPYPQR, IPIQYVL, KVLPVPQK) were present in all cheese samples, regardless of their variant and ripening period. One peptide, RLKKY matching α_S1_-casein, was not identified in the WSEs derived from Gouda after 1 day of ripening—but instead it was found in WSEs after 60 days of ripening. Peptides HPH, VPYPQ, and ARHPHP were present in WSE samples derived from Gouda cheeses analyzed on the first day of ripening. None of them was found in the samples analyzed after 60 days. One peptide, PHQ, was identified only in the WSE samples derived from Gouda with “normative” and “increased” content of β-casein.

**Figure 1:**
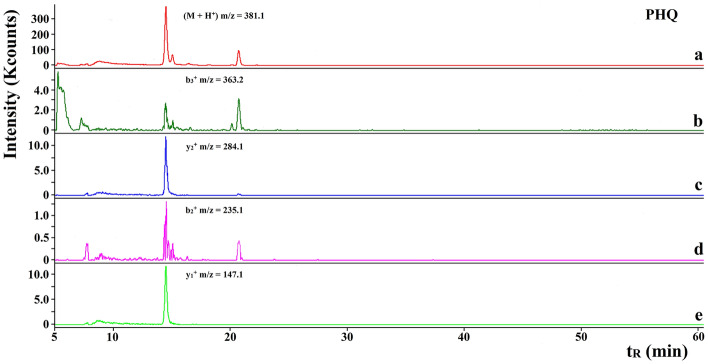
An example of RP-HPLC–MS/MS chromatogram of an antioxidative peptide PHQ, including ion mass/charge ratios, identified in WSEs of Gouda cheese with increased content of β-casein (60th day of ripening). *t*_*R*_ retention time (min).

**Figure 2:**
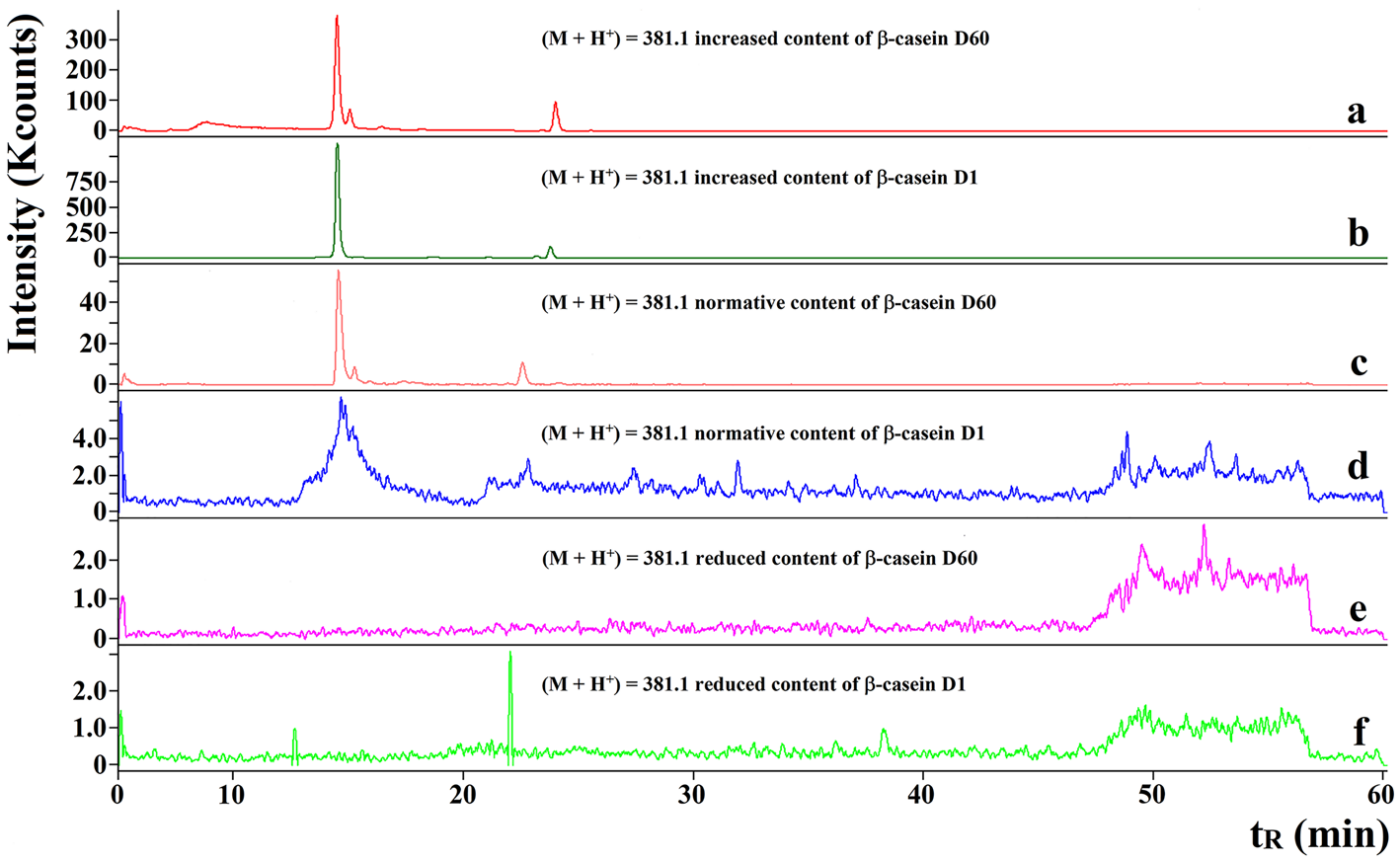
An example of RP-HPLC–MS/MS chromatogram showing the identification of an antioxidative peptide PHQ in WSEs derived from different variants of Gouda cheese. Letters a and b denote samples derived from Gouda cheeses with increased content of β-casein (60th and 1st day of ripening, respectively); letters c and d denote samples derived from Gouda cheeses with normative content of β-casein (60th and 1st day of ripening, respectively); letters e and f denote samples derived from Gouda cheeses with reduced content of β-casein (60th and 1st day of ripening, respectively), *t*_*R*_ retention time (min).

An example of the RP-HPLC–MS/MS chromatogram of a peptide identified in WSE sample is shown in Fig. 1. It presents the antioxidative PHQ peptide (BIOPEP-UWM ID: 8032) found in WSE derived from Gouda with increased content of β-casein (60th day of ripening, see also Fig. 2a). Antioxidative activity of PHQ was introduced by Saito et al.^44^ on the basis of radical- and peroxynitrite-scavenging abilities. Moreover, this sequence has been first described as the component of combinatorial library of tripeptides^44^. PHQ peptide matched the sequence of β-casein (all genetic variants). The m/z (M + H)^+^ of the precursor ion was 381.1 Da. One major peak was observed at ca. 14.5 min. The peptide with the sequence PHQ was identified in the WSE cheese samples derived from: increased content of β-casein and one day of ripening (see Fig. 2b) as well as normative content of β-casein and both stages of ripening (see Fig. 2c,d, respectively). There was no such a sequence in the samples corresponding to Gouda with reduced content of β-casein and both ripening periods (Fig. 2e,f, respectively).

**Figure 3:**
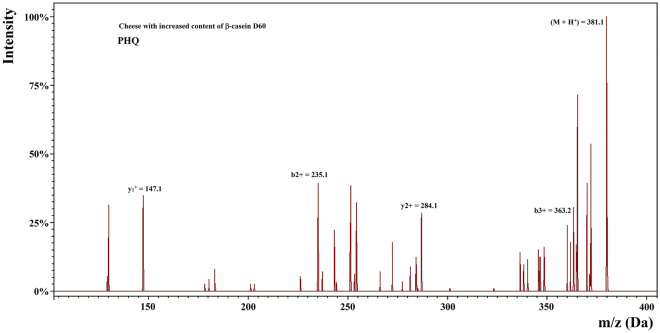
Spectrum of an antioxidative peptide PHQ identified in WSEs of Gouda cheese with increased content of β-casein (60th day of ripening).

The spectrum of the PHQ sequence found in the cheese samples with increased content of β-casein (day 60) is shown in Fig. 3 with the corresponding b/y ion pairs. They were consistent with those predicted theoretically considering the precision of the mass spectrometer used. This method enables identifying peptides composed of 2–5 amino acids when the data on the specificity of enzyme is lacking^23^. Citing the words by Iwaniak et al.^27^ “proteomic software is often unable to detect such peptides using a low resolution ion trap mass spectrometer”.

To recapitulate, regardless of Gouda cheese variant and ripening stage, different peptides matching different fractions of caseins were found in their WSEs. Some antioxidative peptides were generated at both stages of ripening and in all Gouda cheese variants. The general results of the antioxidant assessments (see above) suggest that the antioxidant power of all Gouda cheese variants resulted from the presence of peptides, which was also reported by other scientists^45^. However, some peptidic antioxidants were identified at the end, whereas others at the beginning of ripening, which could explain successive degradation of peptides with extending storage period^45^.

**Figure 4:**
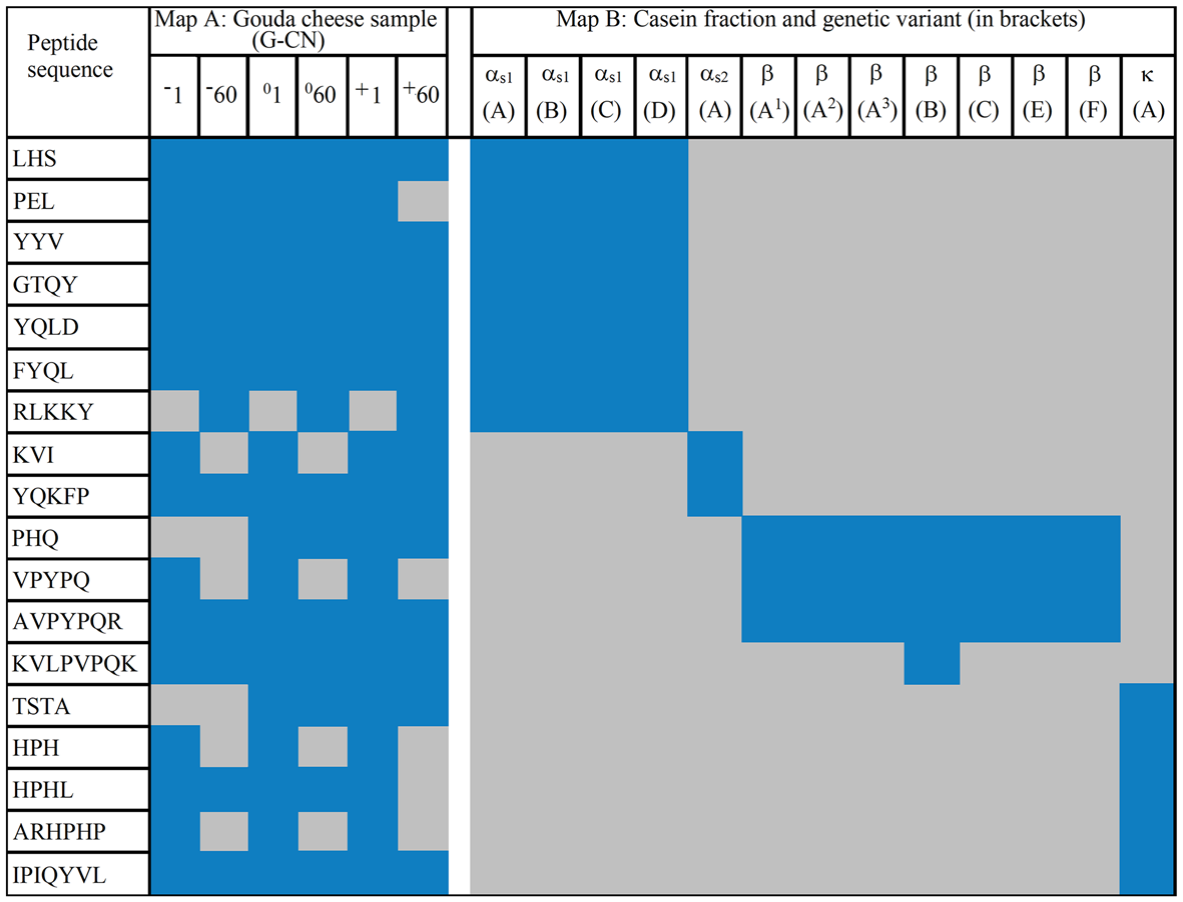
Heatmap of antioxidative peptides identified in Gouda-derived WSEs. Map A: Sample derived from: G-CN^−^_1_, G-CN^−^_60_–G-CN^0^_1_, G-CN^0^_60_, G-CN^+^_1_, G-CN^+^_60_, meaning Gouda cheese with reduced, normative, and increased content of β-casein after the 1st and 60th day of ripening (subscripts: 0 and 60, respectively); Map B: casein fraction the peptide matches to; colors: blue—“yes”, gray—“no”. Figure prepared using the Heatmapper program (see Methods).

Finally, additional in silico predictions were carried out to support the results of peptide identification in WSEs. The first step involved creating the heatmaps to visualize the presence of antioxidative peptides in all analyzed Gouda cheeses and casein sequences. The heatmap is presented in Fig. 4.

**Figure 5:**
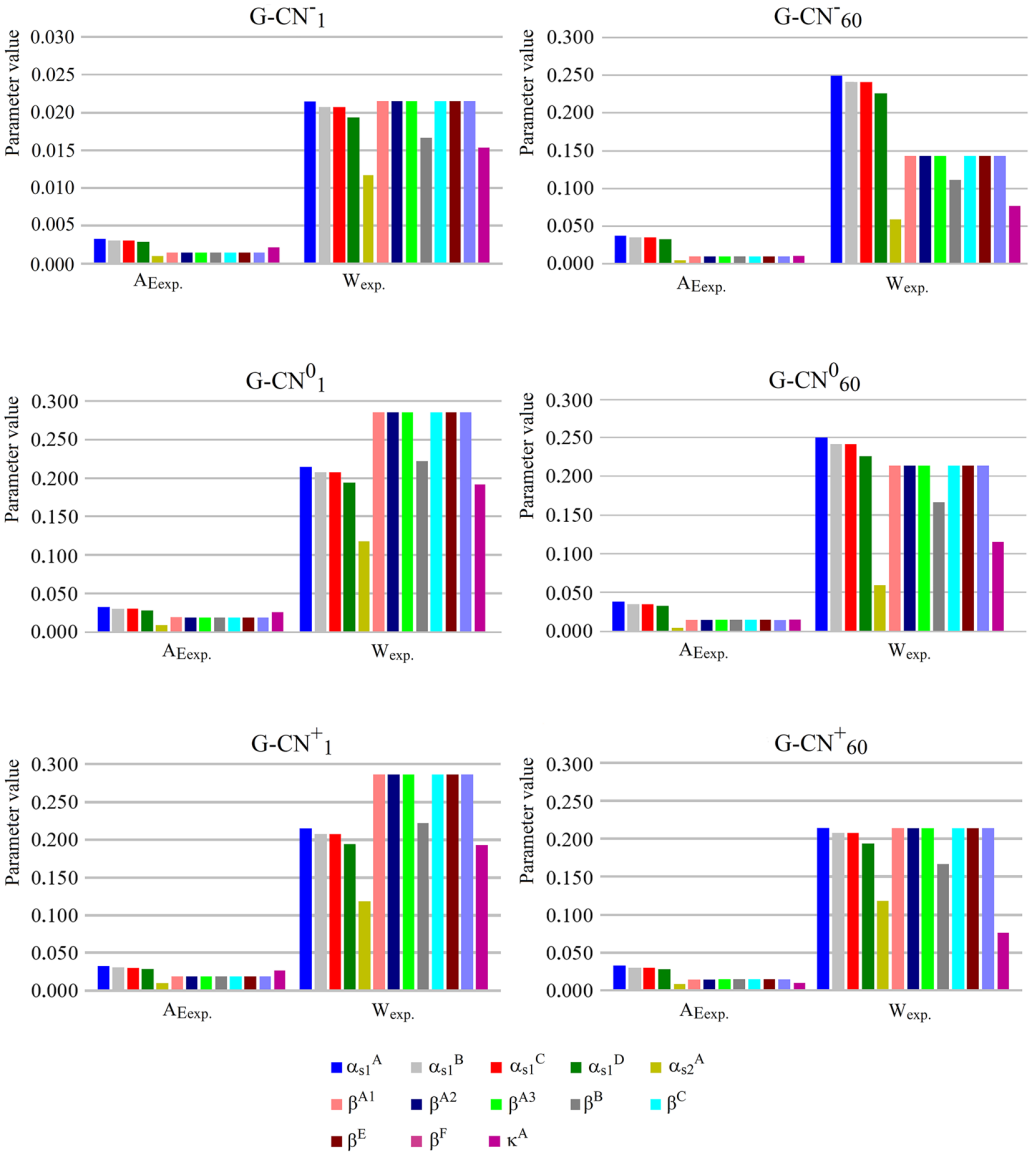
Graphical visualization of A_Eexp_ and W_exp_ values describing the efficiency of the release of antioxidant peptides from Gouda cheese variants. G-CN^−^_1_, G-CN^−^_60_–G-CN^0^_1_, G-CN^0^_60_, G-CN^+^_1_, G-CN^+^_60_, meaning Gouda cheese with reduced, normative, and increased content of β-casein after the 1st and 60th day of ripening (subscripts: 0 and 60, respectively); color bars—casein sequence including its genetic variant (see the superscripts).

Heatmap A shows the presence of antioxidative peptides in individual Gouda cheese-derived WSE samples. The majority of antioxidant peptides were identified in all WSEs (blue area). Three peptides were absent (RLKKY, PHQ, and TSTA; grey area) in the samples derived from G-CN^−^_1_. Moreover, the RLKKY sequence was also absent in the G-CN^0^_1_ and G-CN^+^_1_ samples. On the other hand, 4, 5, and 6 peptides were lost in WSEs derived from G-CN^0^_60_, G-CN^+^_60_ and G-CN^−^_60_, respectively. During cheese ripening, plasmin and LAB-derived (i.e., lactic acid bacteria) enzymes contribute to the production of peptides, which can be accumulated or further degraded during storage^46^. This observation may explain the presence of peptides in Gouda cheese at the beginning of its ripening and their lack in the cheese at the final stage of this process. Heatmap B shows which casein sequences were the best sources of antioxidative peptides identified in WSEs. It was found that the best sources of such peptides were all genetic variants of β- as well as of α_s1_-caseins. These results were consistent with our initial in silico predictions. This consistency of the results concerned the casein sequences being the best sources of antioxidative peptides, but not the number of peptides that were identified using in silico and in vitro protocols. Finally, the results provided by heatmaps enabled calculating the values of the parameters describing proteolysis efficiency^27,47^ namely, the frequency of released fragments with antioxidative activity during Gouda cheese ripening (A_Eexp._), and the relative frequency of release of antioxidative fragments during Gouda cheese ripening (W_exp._). The results are presented in Fig. 5.

The highest frequency of the release of antioxidative peptides (A_Eexp._) was determined for the WSEs obtained from G-CN^−^_60_ and G-CN^0^_60_ samples (A_Eexp._ = 0.038 each). It was related to the efficient release of antioxidative peptides from α_S1_-casein (genetic variant A). The A_Eexp._ referring to the release of antioxidative peptides from the sequence above, but calculated for the G-CN^+^_60_ was 0.032. Identical values of this parameter were obtained for other cheese variants and the same casein sequence. All A_Eexp_ values were related to the number of the antioxidative peptides that were matching the casein sequences (the higher the number, the better the match). Taking into account the relative frequency of release of antioxidative fragments during Gouda cheese ripening (W_exp._), the highest value was determined for the samples derived from G-CN^0^_1_ and G-CN^+^_1_ cheeses (W_exp._ = 0.286 each). This value referred to the relative frequency of the release of antioxidative peptides from β-casein (genetic variants A^1^, A^2^, A^3^, C, E, and F). The W_exp._ values obtained for the same variants of cheese but after 60 days of ripening were 0.214 each. Based on the values of A_Eexp._ and W_exp._, the progress in cheese ripening was found to contribute to the release of antioxidative peptides; however, these peptides were probably the substrates for further hydrolysis. Finally, based on results presented in Figs. 4 and 5, it can be concluded that α_S1_-casein was the major contributor to the antioxidant activity, whereas α_S2_-casein was the least one, regardless of the β-casein content or ripening day.

To summarize, the integrated approach was found useful to analyze different variants of modified Gouda cheese as the sources of antioxidative peptides. This research strategy is consistent with the point of view expressed by Barbano and Lynch^48^, according to whom, the combination and rapid development of different analytical methods for peptide identification in dairy foods would enable better understanding of the processes contributing to the more complex characteristics of nutritional and sensory values of cheeses. These methods can also include bioinformatic-aided characteristics of Gouda cheese derived-peptides^27^. However, it needs to be elucidated that in silico methods that were applied in our study “simplified” some phenomena naturally taking place in a food system, like e.g., ripening cheese. The production of peptides in ripening cheeses is a more complex issue than computer simulations. Apart from the action of coagulant, being the crucial step in cheese production, cheese proteolysis is also the result of the action of plasmin and microbial enzymes. The latter are crucial in the process of cheese ripening. Peptides’ release during cheese ripening depends on the enzyme involved, stage of proteolysis, and rate of hydrolysis of a particular casein fraction^49^. These factors are “not considered” by computer programs, including the BIOPEP-UWM database^15^. For example, a lack of data on the specificity of endogenous proteases was one of the limiting factors of an in silico identification of peptides in caseins. Thus, the detection (or not) of antioxidative peptides in the above protein fractions had to be made using the profile of potential antioxidative activity of casein. Despite such limitations, the usefulness of in silico tools in the analysis of “proteins as the sources of bioactive peptides” was confirmed in several studies^50–54^. To the best of our knowledge, there is no ideal bioinformatic tool for the analysis of food-derived components, including peptides.

To recapitulate, this study is the first which shows the application of integrated approach to study an antioxidative potency of modified dairy product i.e. Gouda cheese. The integrated approach combining in silico and in vitro analyses of Gouda cheese variants with different β-casein content allowed us to identify some regularities concerning the potential of caseins as sources of antioxidative peptides. However, some discrepancies could also be found when comparing the results obtained in silico and in vitro. The possible factors affecting the differences were discussed in our previous works and included, e.g., differences between the numbers of peptides identified in the casein sequences. In silico analyses include, e.g., the repetitions of the motif in a protein sequence, whereas identification of a peptide by RP-HPLC–MS/MS confirms its presence in a sample (if any), but does not show its quantity. All Gouda cheeses exhibited the antioxidative potential which differed depending on the assay used, which shows their potential as antioxidative-promoting food. Additionally, some authors^55^ recommend undertaking further studies involving, e.g., cell culture and/or animal models to get more insights on, e.g., mechanisms of action of food-derived antioxidative agents, their uptake, and metabolism.

## Methods

### Reagents

The following reagents were purchased from Sigma-Aldrich, Sp. z o. o., Poznan, Poland: acetic acid (C_2_H_4_O_2_, catalog No. A6283-1L), tris(hydroxymethyl)aminomethane hydrochloride (TRIS–HCl, cat. No. 93313), 2,2-diphenyl-*β*-picrylhydrazyl (DPPH, catalog No. D9132), 2, 2′-azino-bis(3-ethylbenzotialozline-6-sulfonic acid (ABTS, catalog No. A3219), phosphate buffer saline pH 7.4 (catalog No. P3813), trifluoroacetic acid (TFA, catalog No. T6508), urea (catalog No. U5378), 2,2-bis(hydroxymethyl)-2,2′,2″-nitrilotriethanol (BIS–TRIS, catalog No. B9754), sodium phosphate monobasic (NaH_2_PO_4_, catalog No. P5379), sodium hydroxide (NaOH, catalog No. S8045-500G), sodium phosphate dibasic (Na_2_HPO_4_, catalog No. P8281), potassium ferricyanide (K_3_Fe(CN)_6_, catalog No. 702587), trichloroacetic acid (TCA, catalog No. T6399), ferric chloride hexahydrate (FeCl_3_ × 6H_2_O, catalog No. 236489), iron (II) chloride tetrahydrate (FeCl_2_ × 4H_2_O, catalog No. 380024), ferrozine (TPTZ, 2,4,6-Tris(2-pyridyl)-s-triazine, catalog No. T1253), EDTA (ethylenediaminetetraacetic acid, catalog No. E9884), sodium acetate (CH_3_COONa, catalog No.S8750), sodium dodecyl sulfate Bioreagent (CH_3_(CH_2_)_11_OSO_3_Na, catalog No. L3771), glycerol (HOCH_2_CH(OH)CH_2_OH, catalog No. G5516), glycine (NH_2_CH_2_COOH, catalog No. G8898), Trizma base (NH_2_C(CH_2_OH)_3_, catalog No. T1503), bromphenol blue sodium salt (C_19_H_9_Br_4_NaO_5_S, catalog No. B5525), brilliant blue R (C_45_H_44_N_3_NaO_7_S_2_, catalog No. B7920), dithiothreitol (HSCH_2_CH(OH)CH(OH)CH_2_SH, catalog No. 43819), and acetic acid (glacial) (CH_3_CO_2_H, catalog No. 1018302500). Acetonitrile (ACN, supplier: Romil, Waterbeach, UK, catalog No. ROMILH048L), hydrochloric acid (HCl; supplier: Chempur, Piekary Slaskie, Poland): catalog No. 805313160 (0.1 mol/dm^3^) and 805313164 (1.0 mol/dm^3^), ethanol (C_2_H_5_OH, supplier: Chempur, Piekary Slaskie, Poland, catalogue No. 363-113964200-1L), methanol (CH_3_OH, supplier: Romil, Waterbeach, UK, catalog No. ROMILH409L and supplier: Eurochem BGD Sp. z o.o.), sulfuric acid (H_2_SO_4_, supplier POCH, Poland, catalogue No. 575000115), boric acid (H_3_BO_3_, supplier POCH, Poland, catalogue No. 531360738), sucrose (C_12_H_22_O_11_, supplier POCH, Poland, catalogue No. 772090110), Kjeltabs (K_2_SO_4_ + CuSO_4_·5H_2_O, supplier POL-AURA, Poland, catalogue No. 525085860#100) and sodium hydroxide (NaOH, supplier: Chempur, Piekary Slaskie, Poland): catalogue No. 810953168 (0.01 mol/dm^3^), 810953160 (0.1 mol/dm^3^) and 810953165 (1.0 mol/dm^3^) were acquired from ABChem Olsztyn, Poland. The Mini-Protean TGX Precast Gels (12% 10-well comb, 30 µL/well) were purchased from Bio-Rad Laboratories (Warsaw, Poland). Chemicals used in cheese production included: CaCl_2_ (0.02%, P.P.H. “STANLAB” Sp. J., Lublin, Poland), starter culture (CHN19, Chr. Hansen, Hoersholm, Denmark), and rennet (Chymax M 1000, Chr. Hansen). All reagents were of analytical grade.

Water used to formulate solutions and buffers was prepared using a Milli-Q PLUS system (Millipore Corp., New York, NY, USA). Nylon membrane filters (Whatman^®^, 0.2 μm pore size, catalog No. WHA7402004) were purchased from Sigma-Aldrich Sp. z o.o. (Poznań, Poland) and Munktell-Filtrak 390 grade filters (catalog No. 8.012.120.900) from EQUIMED (Olsztyn, Poland). All chemicals and reagents were of analytical and/or MS grade.

### Bioinformatic predictions

To predict the presence of antioxidant fragments in caseins, the following sequences were acquired from the BIOPEP-UWM database available at: https://biochemia.uwm.edu.pl/biopep-uwm/^15,56^: β—genetic variants: A^1^, A^2^, A^3^, B, C, E, and F (209/ID 1097; 209/ID 1098; 209/ID 1099; 209/ID 1103; 209/ID 1100; 209/ID 1101; 209/ID 1102; 209/ID 1103, respectively) as well as α_S1_—genetic variants: A, B, C, and D (186/ID 1086; 199/ID 1087; 214/ID 1089; 199/ID 1088; 199/ID 1088, respectively); α_S2_—genetic variant A (222/ID 1090); κ—genetic variant A (190/ID 1117). The numbers in brackets mean the length of the protein chain (i.e. number of residues) and the BIOPEP-UWM accession number of casein, respectively. The above-mentioned prediction is called a profile of potential activity of the protein, which is described as the type and the location of a peptide with specific activity in a protein chain^15^. In our studies, the peptide profile of a protein should be understood as the type and the location of antioxidative fragments in the casein sequences. Our predictions excluded the presence of fragments encrypted in signal peptides which are unlikely to be found in milk and hence in dairy products.

The antioxidative peptide profiles of caseins were obtained using a tool called “Analysis” available in the BIOPEP-UWM database^56^. Thus, after entering the BIOPEP-UWM database and clicking the bar called “Proteins” or “Bioactive peptides”, the procedure was as follows (in exact words as provided in this database): Analysis → Profiles of potential biological activity → Select activity (open the bar and select: antioxidative) → Protein database (open the bar and select the protein sequence using its accession ID).

### Production of Gouda cheese with modified β-casein content

**Figure 6:**
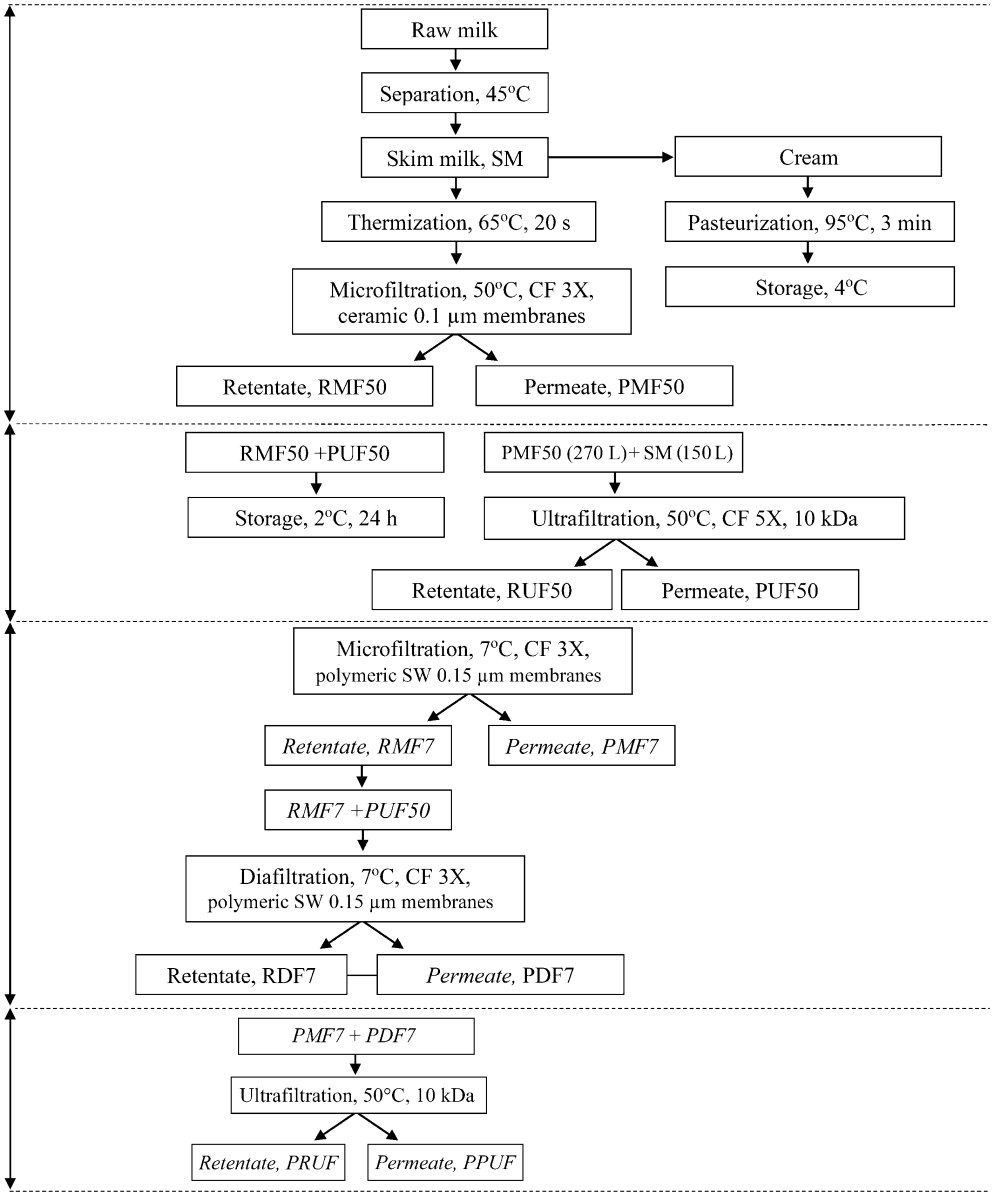
Scheme for production of cheese milk with altered ratio of α_s_-casein to β-casein using a series of filtration processes. *CF* concentration factor, *SM* skim milk, *RMF50* microfiltration retentate produced at 50 °C, *PMF50* microfiltration permeate produced at 50 °C, *RUF50* ultrafiltration retentate produced at 50 °C, *PUF50* ultrafiltration permeate produced at 50 °C, *RMF7* microfiltration retentate produced at 7 °C, *PMF7* microfiltration permeate produced at 7 °C, *RDF7* diafiltration retentate produced at 7 °C, *PDF7* diafiltration permeate produced at 7 °C, *PRUF* ultrafiltration retentate from MF/DF permeates, *PPUF* ultrafiltration permeate from MF/DF permeates, *SW* polyethersulfone spiral-wound membrane.

**Figure 7:**
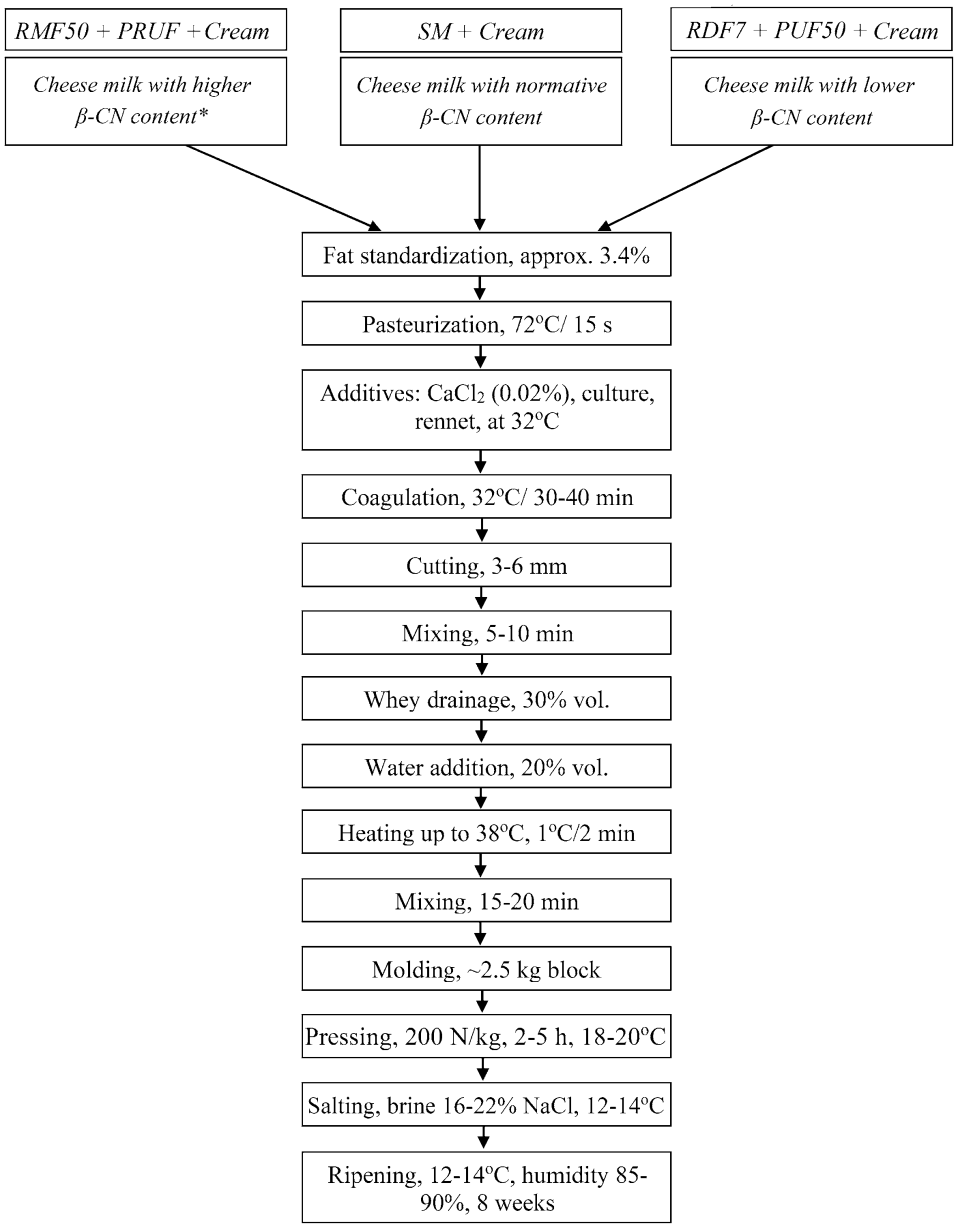
The scheme of Gouda cheese manufacture (abbreviations as in Fig. 6). *The content of β-casein (β-CN) in cheese milk used to produce particular variants of Gouda is shown in Table 1.

The Gouda cheese was produced in a semi-industrial scale at the pilot plant of the University’s Dairy Research and Development Center (Department of Dairy Science and Quality Management, University of Warmia and Mazury in Olsztyn) as described by Iwaniak et al.^27^. The series of membrane filtration processes were applied to modify β-casein content in cheese milk (Fig. 6). The production protocol for Gouda cheese manufacture is shown in Fig. 7. Finally, three variants of Gouda cheese with different contents of β-casein were produced. They were defined as: G-CN^0^, G-CN^+^, G-CN^−^ meaning Gouda cheese with normative, increased, and reduced content of β-casein, respectively. To summarize, there were three productions of each Gouda cheese variant. Thus, the sample should be understood as a mixture of sub-samples collected from the independent batch representing individual variant of Gouda cheese.

The samples of cheese milk were analyzed for total nitrogen (TN) and non-casein nitrogen (NCN) contents using the Kjeldahl method^57^ (method 991.20; 33.2.11), and Kjeldahl method^57^ (method 998.05; 33.2.64) with modifications according to Wojciechowski and Barbano^58^, respectively. Casein was calculated by subtracting NCN from TN and multiplying the result by 6.38.

### Protein composition of cheese milk and monitoring of the proteolysis of Gouda cheese variants using SDS-PAGE electrophoresis

SDS-PAGE was deployed to determine the relative protein proportions (band %) according to the protocol proposed by Zulewska et al.^59^. The change in the content of β-casein in milks used to produce Gouda cheeses was expressed as the ratio of αs-casein to β-casein and the percentage of β-casein of milk proteins in cheese milks used for Gouda production^59^.

The proteolysis of αs- and β-CN during Gouda aging was also monitored by SDS-PAGE. The procedure was described by Verdi et al.^60^, except that a constant 12% acrylamide gel (Biorad Laboratories, Warsaw, Poland) concentration was used. 10 μL of day-1 and day-60 cheese sample buffer mixtures were loaded per slot. As a reference sample, a pasteurized milk was also loaded on each gel at a volume of 7 μL. The destained gels were scanned using a Lumi Bis Bioimaging Systems DNR scanner (Jerusalem, Israel) and analyzed using TotalLab 1D v 11.3 (New Castle, England). The gels were scanned to obtain relative protein proportions within each sample. Each sample was replicated 3 times on a gel. Para-κ-CN was used as an internal standard, since it is not hydrolyzed during Cheddar cheese aging^61^, and similar patterns apply to Gouda cheese. The ratio of α_s_-CN (i.e., αs_1_- + α_s2_-CN) peak height and β-CN peak height to para-κ-CN peak height at each time of aging was calculated^62^. The percentage degradation of α_s_-CN was calculated by subtracting each of the ratios (α_s_-CN/para-κ-CN) on day 60 from the ratio (α_s_-CN/para-κ-CN) on day 1, dividing by the ratio (α_s_-CN/para-κ-CN) on day 1, and multiplying by 100^63^. The same procedure was followed for β-CN degradation. The original electropherograms of cheese samples are presented in Supplementary Figs. 1S and 2S. Gels were obtained within the same experiment and concerned the same samples (cheese) in different time related to their process of ripening.

### Determination of antioxidant activity of water-soluble extracts derived from Gouda cheese

#### General information

Regardless of the assay applied, absorbance of samples derived from each Gouda variant was measured using a UV/Vis spectrophotometer (Genesys™ 150). All measurements were performed in triplicate. The final results of each measurement were converted to the concentrations corresponding to the half-maximal antioxidant inhibition (IC_50_) of WSEs. This conversion was done using GraphPad Prism 5.02 software for Windows^®^^64,65^. The computations were carried out using the following options: “Nonlinear regression” → “Dose–response curves—Inhibition” → “inhibition (log) vs. normalized response—variable slope”. They included standard error (at 95% confidence interval). According to the instructions concerning the estimation of IC_50_^66^, we applied at least five separate WSE concentrations (i.e., sample).

#### Radical scavenging activity assay using 2,2-diphenyl-β-picrylhydrazyl (DPPH)

0.1 mL of an aqueous solution of WSE was mixed with 3.9 mL of a freshly prepared DPPH^.^ ethanol solution (60 μM/dm^3^). Then, the samples were left at a room temperature for 45 min. Afterward, their absorbance was measured at λ = 517 nm. The percentage of DPPH^.^ reduction by the sample was calculated using the formula below^34^:

1 $$\%_{{\text{DPPH}}^{\cdot}{{\text{scavenging rate}}}} = \left[ \left( {\text{A}} - {\text{B}} \right)/{\text{A}} \right] \times 100$$

where: A—absorbance of the control sample (H_2_O + DPPH^.^); B—absorbance of the test sample (WSE + DPPH^.^).

#### Radical scavenging activity assay using 2,2′-azino-bis(3-ethylbenzotialozline-6-sulfonic acid) diammonium salt (ABTS) cation radical

Firstly, the stock solution combined of 5 mL ABTS·^+^ (7 mM) and 88 μL of potassium persulfate (K_2_SO_4_; 140 mM/dm^3^) was prepared and left in the dark at a room temperature for 12–16 h. Then, the ABTS^·+^ solution was diluted with a phosphate buffer saline (PBS; 10 mM; pH 7.4) to get an absorbance 0.7 (± 0.02) at 734 nm. Finally, 1 mL of diluted ABTS^·+^ was mixed with 10 μL of the sample (WSE). The absorbance of all samples was measured after 10 min. The rate (%) of ABTS·^+^ reduction by the WSE was calculated using the following formula^34^:

2 $$\%_{{{\text{ABTS}}}^{\cdot + }{{\text{scavenging rate}}}} = \left[ \left( {\text{A}} - {\text{B}} \right)/{\text{A}} \right] \times 100$$

where: A—absorbance of the control sample (H_2_O + ABTS^·+^); B—absorbance of the test sample (WSE + ABTS^·+^).

#### Fe ion-chelating activity

1 mL of WSE solution was mixed with 0.02 mL of FeCl_2_ × 4H_2_O (2.0 mM/dm^3^) and 3.7 mL of distilled water. The reaction was initiated by adding 0.04 mL of ferrozine 3-(2-pyridyl)-5,6-bis (4-phenyl-sulfonic acid)-1,2,4-triazine). Then, the samples were incubated at a room temperature for 20 min. Finally, the absorbance of the mixture was measured at 562 nm against a blank. EDTA (ethylenediaminetetraacetic acid) was used as a positive control. The Fe ion-chelating percentage was calculated using the following equation^67^:

3 $$\%_{{{\text{Fe ion}} - {\text{chelating activity}}}} = \left[ {\left( {{\text{A}}_{0} - {\text{A}}_{{1}} } \right)/{\text{A}}_{0} } \right] \times 100$$

where: A_0_—absorbance without the sample (water instead), A_1_—absorbance of the chelator (sample; WSE).

#### Ferric-reducing antioxidant power (FRAP)

The FRAP reagent (1150 µL per sample) composition was as follows: one part of 10 mM TPTZ, one part of 20 mM FeCl_3_ × 6H_2_O, and ten parts of 300 mM acetate buffer (pH 3.6), at 37 °C. Then, freshly prepared FRAP reagent was mixed with different concentrations of WSE methanolic solutions (50 µL) and incubated (20 min; room temperature). Finally, the absorbance of all samples was measured at λ = 593 nm against a blank (1150 µL FRAP reagent + 50 µL distilled water)^68^. The percentage of the ferric-reducing antioxidant power (FRAP) was calculated using the formula adapted from Venskutonis et al.^69^:

4 $$\%_{{{\text{FRAP}}}} = \left[ {\left( {{\text{B}} - {\text{A}}} \right)/{\text{B}}} \right] \times 100$$

where: A—absorbance of the blank (see above); B—absorbance of the test sample (see above).

#### Identification of antioxidative peptides in water-soluble extracts of Gouda cheese variants using liquid chromatography and mass spectrometry (RP-HPLC–MS/MS) analysis

The identification of peptides in WSEs was carried out using the Reversed Phase High Performance Liquid Chromatography (RP-HPLC–MS/MS, i.e. RP-HPLC coupled with mass spectrometry). The data concerning the equipment and software applied for data acquisition and processing was described by Iwaniak et al.^27^. Details concerning LC–MS/MS analysis are presented in Table 4.

**Table 4:** Conditions of RP-HPLC–MS/MS analysis^27^.

Feature	Description		
Column	Jupiter Proteo Phenomenex^®^ (Torrance, CA, USA) 250 × 2 mm, particle diameter—4 μm, pore diameter—90 Å		
Solvent A	0.01% (v/v) of TFA in water		
Solvent B	0.01% (v/v) of TFA in acetonitrile		
Flow rate	0.2 mL/min		
Column temperature	40 °C		
Injection volume	10 μL		
HPLC gradient	0.00–40.00 min	0–40% B	Peptide separation
	40.01–45.00 min	40–100% B	Column washing
	45.01–50.00 min	100% B	
	50.01–51.00 min	100–0% B	Column conditioning
	51.01–60.00 min	0% B	
Data acquisition period	5–60 min		
Needle voltage	6000 V		
Shield voltage	600 V		
Positive polarity with current ionization	600 V		
Capillary voltage	100 V		
Spraying and drying gas pressure (nitrogen)	55 and 30 psi, respectively		
Drying gas temperature	390 °C		
Flow rate of damping gas (helium)	0.8 mL/min		
Mass to charge ratio range	100–2000 Da		
Frequency of data recording	0.05–0.07 Hz		
Number of microscans per scan	5		
Isolation window	3 Da		

The presence of antioxidative peptides in all WSEs was visualized using the Heatmapper program (http://www.heatmapper.ca/)^70,71^. Additionally, we calculated the frequency of the release of antioxidative fragments during cheese ripening (A_Eexp_.) and the relative frequency of the release of bioactive peptides during cheese ripening (W_exp_.)^27^. The equations describing these two parameters were as follows:

5 $${\text{A}}_{{{\text{Eexp}}.}} = {\text{ d}}_{{{\text{exp}}.}} /{\text{N}}$$

where: d_exp._—the number of antioxidative peptides identified in WSEs depending on the stage of Gouda ripening; N—the number of amino acid residues in a protein (acquired from the BIOPEP-UWM database).

6 $${\text{W}}_{{{\text{exp}}}} = {\text{ A}}_{{{\text{Eexp}}.}} /{\text{A}}$$

where: A—the frequency of the occurrence of antioxidative peptides in a protein sequence (acquired from the BIOPEP-UWM database). These formulas were analogical to those introduced by Minkiewicz et al.^47^.

### Statistical analysis

To determine whether there were significant differences in the proportion of α_s_-CN to β-CN between Gouda cheese variants, all data were analyzed by ANOVA using Statistica (version 13.1, 1984–2016, StatSoft Inc., Tulsa, OK). *P* value < 0.05 was considered significant in all tests.

## Supplementary Information

Supplementary Information.

## Data Availability

All data are available within this article as well as Supplementary Materials.
