# Supplementary material for: An integrated approach to the analysis of antioxidative peptides derived from Gouda cheese with a modified β-casein content
Source: Sci Rep. 2022 Aug 3;12:13314. doi: 10.1038/s41598-022-17641-x (PMC9349201; doi:10.1038/s41598-022-17641-x)
Supplement: Supplementary file 1 — Supplementary Information. [file 41598_2022_17641_MOESM1_ESM.pdf]

Supplementary Materials

An integrated approach to the analysis of antioxidative peptides derived from Gouda cheese with a modified *β*-casein content

Anna Iwaniak*[1], Damir Mogut[1], Piotr Minkiewicz[1], Justyna Żulewska[2], Małgorzata Darewicz[1]

[1] University of Warmia and Mazury in Olsztyn, Faculty of Food Science, Chair of Food Biochemistry, Pl. Cieszyński 1, 10-719 Olsztyn-Kortowo, Poland

[2] University of Warmia and Mazury in Olsztyn, Faculty of Food Science, Department of Dairy Science and Quality Management, Oczapowskiego 7, 10-719 Olsztyn-Kortowo, Poland

* ami@uwm.edu.pl

Supplementary Tables

Table 1S. *In silico* results of the presence of antioxidative motifs in casein sequences (based on data acquired from the BIOPEP-UWM database[56]).

	Location of antioxidative sequence in casein chains*												
Peptide	α_{S1} (A)**	α_{S1} (B)	α_{S1} (C)	α_{S1} (D)	α_{S2} (A)	β (A^1)	β (A^2)	β (A^3)	β (B)	β (C)	β (E)	β (F)	κ (A)
LH	107-108	120-121	120-121	135-136	-	133-134	133-134	133-134	133-134	133-134	133-134	133-134	-
HPHL	-	-	-	-	-	-	-	-	-	-	-	-	121-124
PYY	-	-	-	-	-	-	-	-	-	-	-	-	80-82
HPH	-	-	-	-	-	-	-	-	-	-	-	-	119-121<br>121-123
HL	-	-	-	-	-	134-135	134-135	134-135	134-135	134-135	134-135	134-135	123-124
VKEAMAPK	-	-	-	-	-	98-105	98-105	98-105	98-105	98-105	98-105	98-105	-
ARHPHPHLSF M	-	-	-	-	-	-	-	-	-	-	-	-	117-127
AY	130-131<br>145-146	143-144<br>158-159	143-144<br>158-159	158-159<br>173-174	-	-	-	-	-	-	-	-	-
LY	-	-	-	-	114-115	192-193	192-193	192-193	192-193	192-193	192-193	192-193	-
AVPYPQR	-	-	-	-	-	177-183	177-183	177-183	177-183	177-183	177-183	177-183	-
KVLPVPQK	-	-	-	-	-	-	-	-	169-176	-	-	-	-
VLPVPQK	-	-	-	-	-	-	-	-	170-176	-	-	-	-
YQEP	-	-	-	-	-	-	-	-	193-196	-	-	-	-
YQEPVLGP	-	-	-	-	-	-	-	-	193-200	-	-	-	-
YFYPEL	131-136	144-149	144-149	159-164	-	-	-	-	-	-	-	-	-
EL	26-27<br>128-129<br>135-136	39-40<br>141-142<br>148-149	39-40<br>141-142<br>148-149	54-55<br>156-157<br>163-164	-	2-3<br>5-6<br>44-45	2-3<br>5-6<br>44-45	2-3<br>5-6<br>44-45	2-3<br>5-6<br>44-45	2-3<br>5-6<br>44-45	2-3<br>5-6<br>44-45	2-3<br>5-6<br>44-45	-
PYPQ	-	-	-	-	-	179-182	179-182	179-182	179-182	179-182	179-182	179-182	-
WY	151-152	164-165	164-165	179-180	-	-	-	-	-	-	-	-	-
YYA	-	-	-	-	-	-	-	-	-	-	-	-	81-83
YYV	152-154	165-167	165-167	180-182	-	-	-	-	-	-	-	-	-
YYQ	-	-	-	-	-	-	-	-	-	-	-	-	63-65

YLY	-	-	-	-	113-115	-	-	-	-	-	-	-	-
WYY	151-153	164-166	164-166	179-181	-	-	-	-	-	-	-	-	-
YFY	131-133	144-146	144-146	159-161	-	-	-	-	-	-	-	-	-
NYY	-	-	-	-	-	-	-	-	-	-	-	-	62-64
LHL	-	-	-	-	-	133-135	133-135	133-135	133-135	133-135	133-135	133-135	-
LHS	107-109	120-122	120-122	135-137	-	-	-	-	-	-	-	-	-
PHL	-	-	-	-	-	-	-	-	-	-	-	-	122-124
PHQ	-	-	-	-	-	147-149	147-149	147-149	147-149	147-149	147-149	147-149	-
PWD	-	-	-	-	123-125	-	-	-	-	-	-	-	-
PWI	-	-	-	-	207-209	-	-	-	-	-	-	-	-
KVI	-	-	-	-	214-216	-	-	-	-	-	-	-	-
KD	29-30	42-43	42-43	57-58	-	-	-	-	-	-	-	-	34-35
PEL	134-136	147-149	147-149	162-164	-	-	-	-	-	-	-	-	-
YPEL	133-136	146-149	146-149	161-164	-	-	-	-	-	-	-	-	-
FYPEL	132-136	145-149	145-149	160-164	-	-	-	-	-	-	-	-	-
IQY	-	-	-	-	-	-	-	-	-	-	-	-	49-51
YVL	-	-	-	-	-	-	-	-	-	-	-	-	51-53
FALPQYLK	-	-	-	-	189-196	-	-	-	-	-	-	-	-
PYVRYL	-	-	-	-	217-222	-	-	-	-	-	-	-	-
PW	-	-	-	-	123-124<br>207-208	-	-	-	-	-	-	-	-
IR	-	-	-	-	-	-	-	-	-	-	-	-	30-31
LK	88-89	101-102	101-102	116-117	179-180<br>195-196	-	-	-	-	-	-	-	-
KP	-	-	-	-	206-207	-	-	-	-	-	-	-	67-68<br>84-85
TY	-	-	-	-	34-35	-	-	-	-	-	-	-	-

TSTA	-	-	-	-	-	-	-	-	-	-	-	-	186-189
VY	-	-	-	-	198-199	59-60	59-60	59-60	59-60	59-60	59-60	59-60	-
VPYPQ	-	-	-	-	-	178-182	178-182	178-182	178-182	178-182	178-182	178-182	-
IPIQYVL	-	-	-	-	-	-	-	-	-	-	-	-	47-53
GPVRGPFPII	-	-	-	-	-	199-208	199-208	199-208	199-208	199-208	199-208	199-208	-
AW	150-151	163-164	163-164	178-179	-	-	-	-	-	-	-	-	-
LW	185-186	198-199	198-199	213-214	-	-	-	-	-	-	-	-	-
YAKPA	-	-	-	-	-	-	-	-	-	-	-	-	82-86
GTQY	157-160	170-173	170-173	185-188	-	-	-	-	-	-	-	-	-
ARHPHP	-	-	-	-	-	-	-	-	-	-	-	-	117-122
RHPHP	-	-	-	-	-	-	-	-	-	-	-	-	118-122
RYPS	-	-	-	-	-	-	-	-	-	-	-	-	55-58
SRYPS	-	-	-	-	-	-	-	-	-	-	-	-	54-58
AYPS	145-148	158-161	158-161	173-176	-	-	-	-	-	-	-	-	-
YAKP	-	-	-	-	-	-	-	-	-	-	-	-	82-85
RYQ	-	-	-	-	185-187	-	-	-	-	-	-	-	-
VLSRYPS	-	-	-	-	-	-	-	-	-	-	-	-	52-58
TIASGEP	-	-	-	-	-	-	-	-	-	-	-	-	145-151
YQLD	141-144	154-157	154-157	169-172	-	-	-	-	-	-	-	-	-
GYLEQ	80-84	93-97	93-97	108-112	-	-	-	-	-	-	-	-	-
RDMPIQ	-	-	-	-	-	183-188	183-188	183-188	183-188	183-188	183-188	183-188	-
YPELF	133-137	146-150	146-150	161-165	-	-	-	-	-	-	-	-	-
RLKKY	87-91	100-104	100-104	115-119	-	-	-	-	-	-	-	-	-
YLKT	-	-	-	-	194-197	-	-	-	-	-	-	-	-
TVYQ	-	-	-	-	197-200	-	-	-	-	-	-	-	-
LLR	85-87	20-22<br>98-100	20-22<br>98-100	35-37<br>113-115	-	-	-	-	-	-	-	-	-
YGLN	-	-	-	-	-	-	-	-	-	-	-	-	59-62
YQKFP	-	-	-	-	104-108	-	-	-	-	-	-	-	-

YQL	141-143	154-156	154-156	169-171	-	-	-	-	-	-	-	-	-
FYQL	140-143	153-156	153-156	168-171	-	-	-	-	-	-	-	-	-
NEN	-	17-19	17-19	32-34	-	-	-	-	-	-	-	-	-
KKY	89-91	102-104	102-104	117-119	-	-	-	-	-	-	-	-	-
YLGY	78-81	91-94	91-94	106-109	-	-	-	-	-	-	-	-	-
HIQKEDVPSER	67-77	80-90	80-90	95-105	-	-	-	-	-	-	-	-	-

*Location in a protein precursor sequence; **Genetic variant of casein (in brackets)

Table 2S. Identification of peptides with antioxidative bioactivity in WSEs derived from Gouda cheese with modified β-casein content.

Sequence	Presence in WSE derived from:						[2]CN source	[3]t_R (min)	[1]$(M+H)^+$ (m/z)
	[1]G-CN$^{-}_{1}$	G-CN$^{-}_{60}$	G-CN$^{0}_{1}$	G-CN$^{0}_{60}$	G-CN$^{+}_{1}$	G-CN$^{+}_{60}$			
LHS	+	+	+	+	+	+	α_{S1}	31.556	356.2
PEL	+	+	+	+	+	-	α_{S1}	22.327	358.2
KVI	+	-	+	-	+	+	α_{S2}	9.171	359.3
TSTA	-	-	+	+	+	+	κ	22.986	379.2
PHQ	-	-	+	+	+	+	β	14.539	381.2
HPH	+	-	+	-	+	-	κ	6.554	390.2
YYV	+	+	+	+	+	+	α_{S1}	37.874	444.2
GTQY	+	+	+	+	+	+	α_{S1}	23.777	468.2
HPHL	+	+	+	+	+	-	κ	25.913	503.3
YQLD	+	+	+	+	+	+	α_{S1}	18.878	538.2
FYQL	+	+	+	+	+	+	α_{S1}	23.002	570.3
VPYPQ	+	-	+	-	+	-	β	17.694	603.3
YQKFP	+	+	+	+	+	+	α_{S2}	35.540	682.4
RLKKY	-	+	-	+	-	+	α_{S1}	31.532	707.5
ARHPHP	+	-	+	-	+	-	κ	52.565	714.4
AVPYPQR	+	+	+	+	+	+	β	24.422	830.4
IPIQYVL	+	+	+	+	+	+	κ	19.063	845.5
KVLPVPQK	+	+	+	+	+	+	β	30.743	908.6

[1]G-CN$^{0}_{1}$, G-CN$^{0}_{60}$, G-CN$^{+}_{1}$, G-CN$^{+}_{60}$, G-CN$^{-}_{1}$, G-CN$^{-}_{60}$ - Gouda cheese with normative, increased, and reduced content β-casein after the 1st and 60th day of ripening (subscripts: 0 and 60, respectively); [2]CN-casein; [3]t_R – retention time; [4]$(M+H)^+$ - precursor ion type.

Supplementary Figures

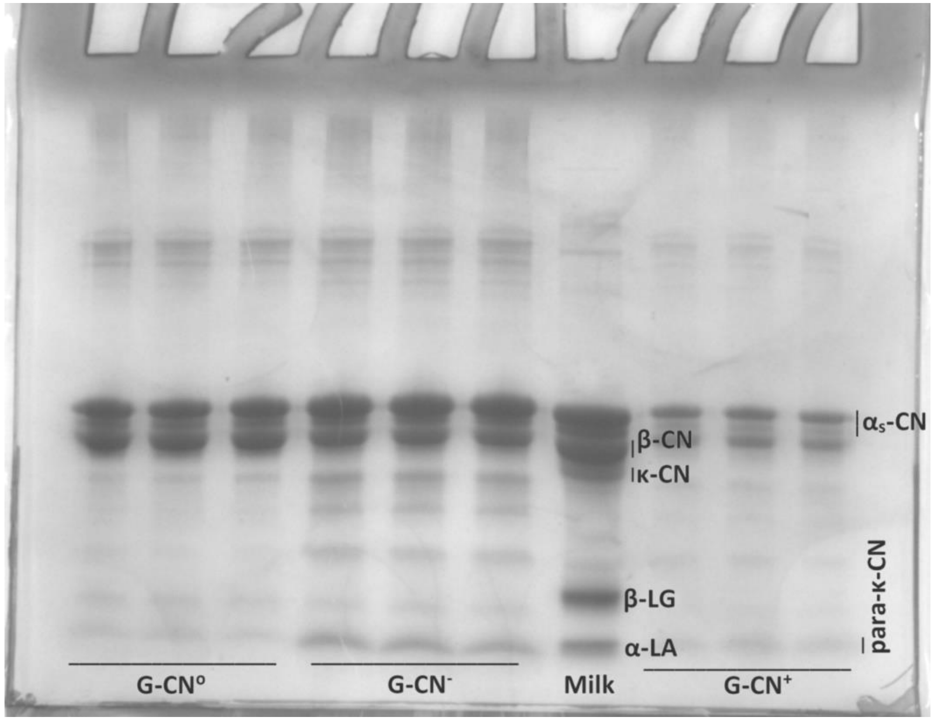

Fig. 1S. SDS PAGE pattern of caseins in Gouda cheese at day 1.

Abbreviations: α_s-CN - α_s-casein; β-CN - β-casein; κ-CN - κ-casein; para-κ-CN - para-κ-casein; β-LG- β-lactoglobulin; G-CN^0, G-CN^-, G-CN^+ - Gouda cheese with normative, reduced, and increased content of β-casein, respectively.

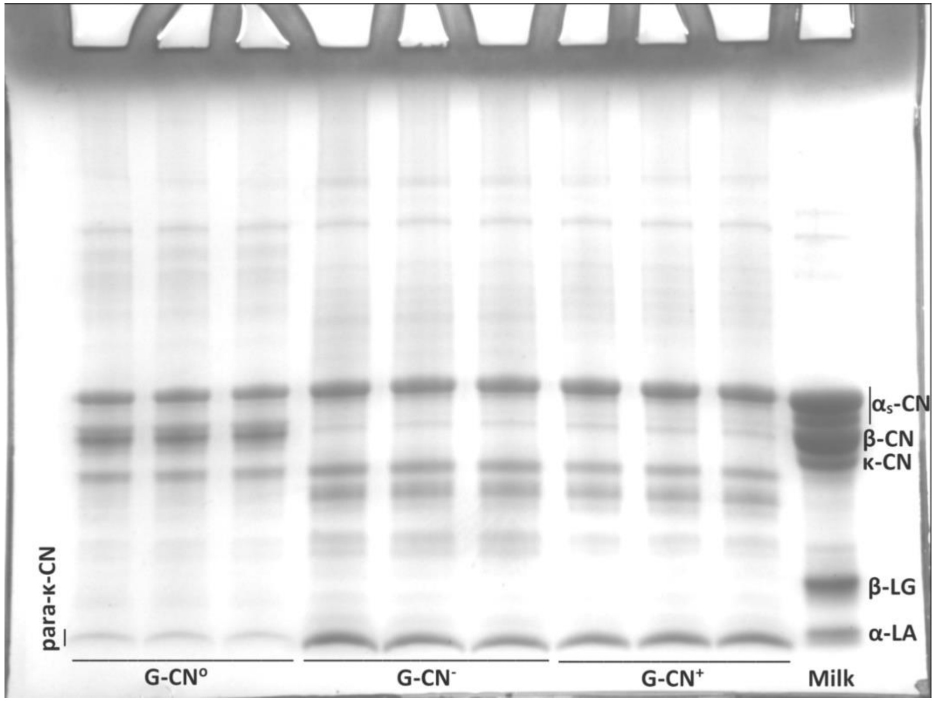

Fig. 2S. SDS PAGE pattern of caseins in Gouda cheese at day 60.

Abbreviations: α_s-CN - α_s-casein; β-CN - β-casein; κ-CN - κ-casein; para-κ-CN - para-κ-casein; β-LG - β-lactoglobulin; α-LA - α-lactalbumin; G-CN^0, G-CN^-, G-CN^+ - Gouda cheese with normative, reduced, and increased content of β-casein, respectively.
